# Supplementary material for: Causal Relationship between Aging and Anorexia Nervosa: A White-Matter-Microstructure-Mediated Mendelian Randomization Analysis
Source: Biomedicines. 2024 Aug 16;12(8):1874. doi: 10.3390/biomedicines12081874 (PMC11351342; doi:10.3390/biomedicines12081874)
Supplement: Supplementary file 1 [file biomedicines-12-01874-s001.zip › Supplementary Figure/Figure S4/Singe SNP plot of MR analysis for TL to ubm-b-2110.pdf]

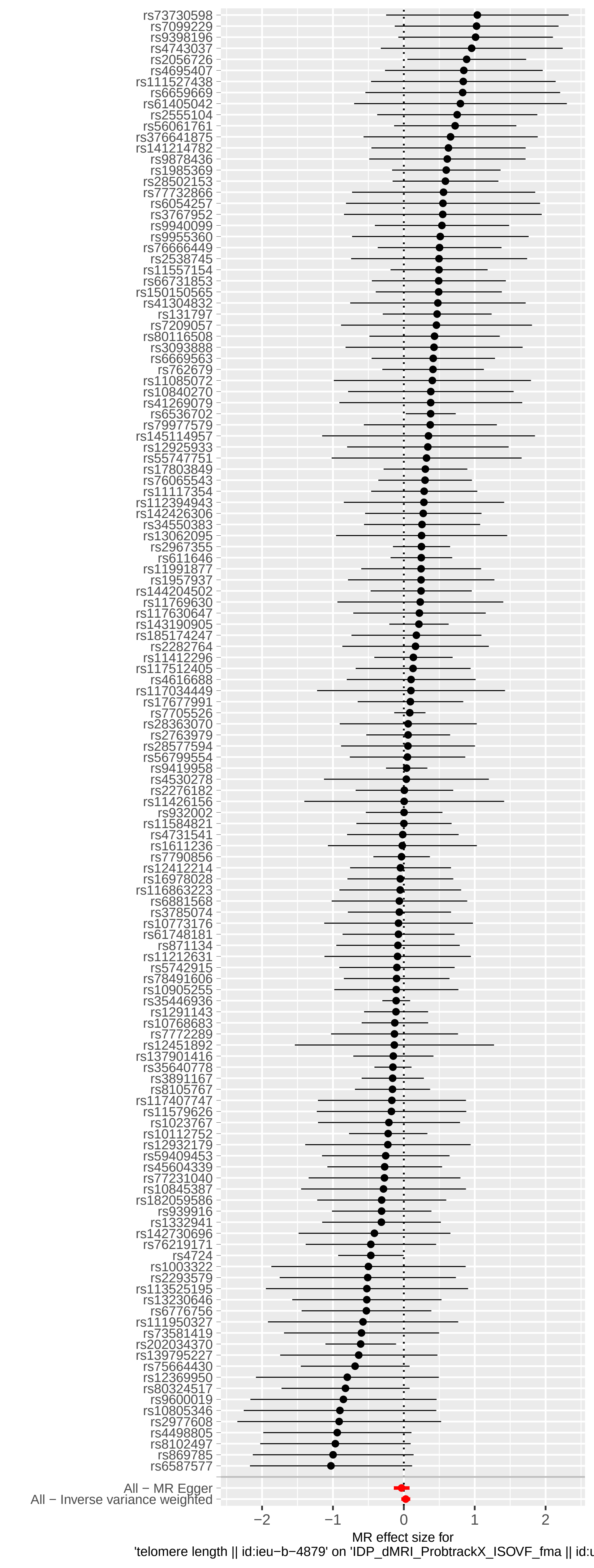
rs73730598
rs7099229
rs9398196
rs4743037
rs2056726
rs4695407
rs111527438
rs6659669
rs61405042
rs2555104
rs56061761
rs376641875
rs141214782
rs9878436
rs1985369
rs28502153
rs77732866
rs6054257
rs3767952
rs9940099
rs9955360
rs76666449
rs2538745
rs11557154
rs66731853
rs150150565
rs41304832
rs131797
rs7209057
rs80116508
rs3093888
rs6669563
rs762679
rs11085072
rs10840270
rs41269079
rs6536702
rs79977579
rs145114957
rs12925933
rs55747751
rs17803849
rs76065543
rs11117354
rs112394943
rs142426306
rs34550383
rs13062095
rs2967355
rs611646
rs11991877
rs1957937
rs144204502
rs11769630
rs117630647
rs143190905
rs185174247
rs2282764
rs11412296
rs117512405
rs4616688
rs117034449
rs17677991
rs7705526
rs28363070
rs2763979
rs28577594
rs56799554
rs9419958
rs4530278
rs2276182
rs11426156
rs932002
rs11584821
rs4731541
rs1611236
rs7790856
rs12412214
rs16978028
rs116863223
rs6881568
rs3785074
rs10773176
rs61748181
rs871134
rs11212631
rs5742915
rs78491606
rs10905255
rs35446936
rs1291143
rs10768683
rs7772289
rs12451892
rs137901416
rs35640778
rs3891167
rs8105767
rs117407747
rs11579626
rs1023767
rs10112752
rs12932179
rs59409453
rs45604339
rs77231040
rs10845387
rs182059586
rs939916
rs1332941
rs142730696
rs76219171
rs4724
rs1003322
rs2293579
rs113525195
rs13230646
rs6776756
rs111950327
rs73581419
rs202034370
rs139795227
rs75664430
rs12369950
rs80324517
rs9600019
rs10805346
rs2977608
rs4498805
rs8102497
rs869785
rs6587577
All − MR Egger
All − Inverse variance weighted
−2
−1
0
1
2
MR effect size for
'telomere length || id:ieu−b−4879' on 'IDP_dMRI_ProbtrackX_ISOVF_fma || id:u
